# The evolution of mosquito baiting: from chemical and electronic methods toward AI

**DOI:** 10.1186/s13071-026-07413-1

**Published:** 2026-05-26

**Authors:** Mustafa Mohammad Shaky, Rohan Reddy Kalavakonda, Sumaiya Afroz Mila, Ankan Ghosh, Michael Futo, Jeremiah Hidayat, Swarup Bhunia, Sandip Ray, Bianca C. Burini

**Affiliations:** 1 Department of Electrical and Computer Engineering, University of Florida, Gainesville, FL 32611 USA; 2 Florida Medical Entomology Laboratory, University of Florida–IFAS, Vero Beach, FL 32962 USA

**Keywords:** Mosquito baits, Organic baits, Chemical baits, Electronic baits, Smart technology, Trap

## Abstract

The spread of mosquito-borne diseases is accelerating owing to several factors, including human-made ones. In response, scientists are developing various types of baits, including organic, chemical, and electronic, to lure mosquitoes and monitor, control, and kill them. These baits function by replicating stimuli that attract mosquitoes, including human breath, body heat, color, and mosquito wingbeat sounds. Wingbeat sound baits generated from the wings of female mosquitoes are particularly effective in attracting males. Carbon dioxide is a potent attractant and the closest human mimic among the available mimics. Human metabolism, pregnancy, skin temperature, microbiomes, and other factors all play significant roles in the attractiveness of humans to mosquitoes. Effective bait design requires a comprehensive understanding of mosquito behavior, including feeding, mating, and oviposition patterns. Lab, semifield, and field experimental studies allow researchers to isolate behavioral responses to specific stimuli before evaluating their effectiveness under more realistic ecological conditions. Optimizing the baiting system power maximizes efficiency and success in baiting mosquitoes. The future of mosquito bait lies in automating and digital twinning of baiting systems and in prioritizing human and environmental safety.

**Graphical Abstract:**

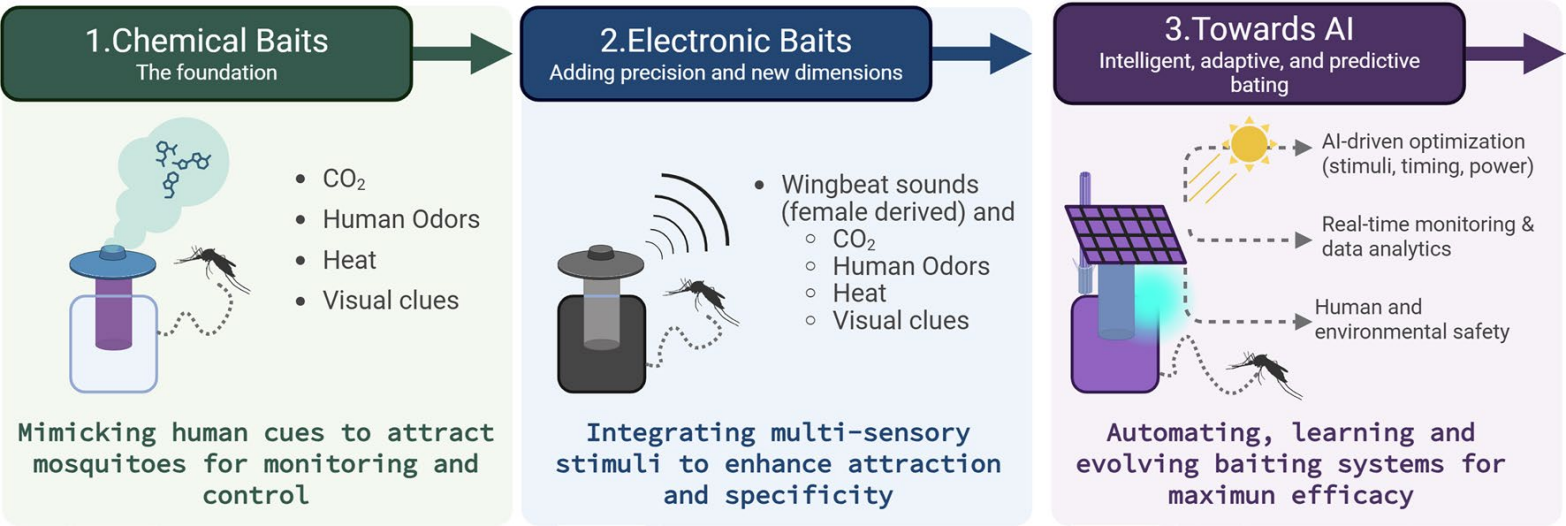

## Background 

Mosquitoes transmit devastating diseases that threaten global health. These include malaria, dengue, Zika, chikungunya, yellow fever, and West Nile virus, among others [1, 2]. Dengue alone infects an estimated 100–400 million people annually, with more than 14 million reported cases and nearly 12,000 deaths in 2024 [3]. Malaria, however, causes the greatest mortality, with approximately 597,000 deaths each year and 263 million cases, predominantly in Sub-Saharan Africa [4].

The public health burden is immense; nearly half of the global population is at risk of mosquito-borne diseases [1]. Outbreaks are occurring with greater frequency, seasonal transmission patterns are shifting, and previously unaffected regions are reporting cases as mosquito ranges expand owing to climate change and rapid urbanization [5]. The economic toll is equally severe, with mosquito-borne diseases imposing an estimated global cost of US $12 billion annually [6].

The threat is worsening owing to mosquitoes developing resistance to insecticides, unplanned expansions of cities and urban landscapes, as well as pathogen resistance to treatments, and persistent difficulties in vaccine development [7–10]. These realities underscore the urgent need for innovative solutions, including genetic control strategies and the integration of artificial intelligence (AI) and Internet of Things (IoT) technologies to enhance surveillance and reduce disease transmission. Consequently, it led to an increased interest in utilizing mosquito sensory biology to aid in tracking, attracting, and controlling them through various combinations of baits [10–21]. Carbon dioxide (CO_2_), oviposition volatiles, host–odor volatile organic compounds (VOCs), humidity, heat, visual contrast, acoustic wingbeat tones, and so on demonstrate effectiveness as baits or attractants [10–21], and the use and application of these baits were analyzed over many decades to understand their effectiveness.

Several reviews have summarized various methods for monitoring, attracting, and trapping mosquitoes, including synthetic mimics, odor mixtures, oviposition signals, bait stations, and smart technologies, as outlined in Table 1 [10–21]. These studies also describe mosquito behavior under certain monitoring and surveillance conditions, as well as the effectiveness of the traps across a range of mosquito species [10–21]. Several review studies also highlight that combining chemical lures with technology could improve mosquito control and surveillance [10]. However, these studies lack analyses on how to effectively utilize these baiting or trapping methods, the connection between the mosquito physiology and bait designs, energy and power optimization, research trends and costs, laboratory, semifield, and field conditions to perform the experiments, the effects on the ecosystem, and safety considerations.

**Table 1:** .

Topic	Keywords	Reference
Smart technology for mosquito control	Artificial intelligence, Smart sensors, Mosquito control, Vector-borne disease	Rajak et al. [10]
Human attractiveness	*Culicidae*, Genetics, Human attractiveness, Microbiota, Mosquito, Mosquito bites	Ellwanger et al. [11]
Malaria vector Lures	Efficacy, mosquito lures, chemo-attractants, odorants, baits, mass trapping, malaria	Watentena et al. [12]
Mosquito olfaction and attraction	Mosquito, attraction, olfaction, kairomones, blood-feeding	Keswani et al. [13]
Mosquito Control	Mosquito control, SIT, *Wolbachia*, ATSB, Transgenics, Gene drive, RIDL, Laser, Conventional strategies	Kumar et al. [14]
	Vector control; *Aedes*; integrated vector management; field trial; insecticide; Europe	Baldacchino et al. [15]
ATSB Baits	Attractive toxic sugar baits, Mosquito control, Sugar feeding	Fiorenzano et al. [16]
	Attractive toxic sugar baits (ATSB); vector control; India; sugar	Kumar et al. [17]
Effect of chemical cues on gravid mosquitoes	*Culicidae*, oviposition, infochemicals, olfactory cues, mosquito behavior, surveillance, control	Mwingira et al. [18]
Semiochemical & Synthetic Attractants	Mosquito, volatile, attractant, compound, host odor;	Dormont et al. [19]
	Malaria, Vector mosquitoes, *Anopheles, Aedes, Culex*, Mosquito life-cycle, Semiochemicals, Chemical communication	Wooding et al. [20]
	*Aedes* spp; Attractant; Lactic Acid	Mubarak et al. [21]

**Fig. 1:**
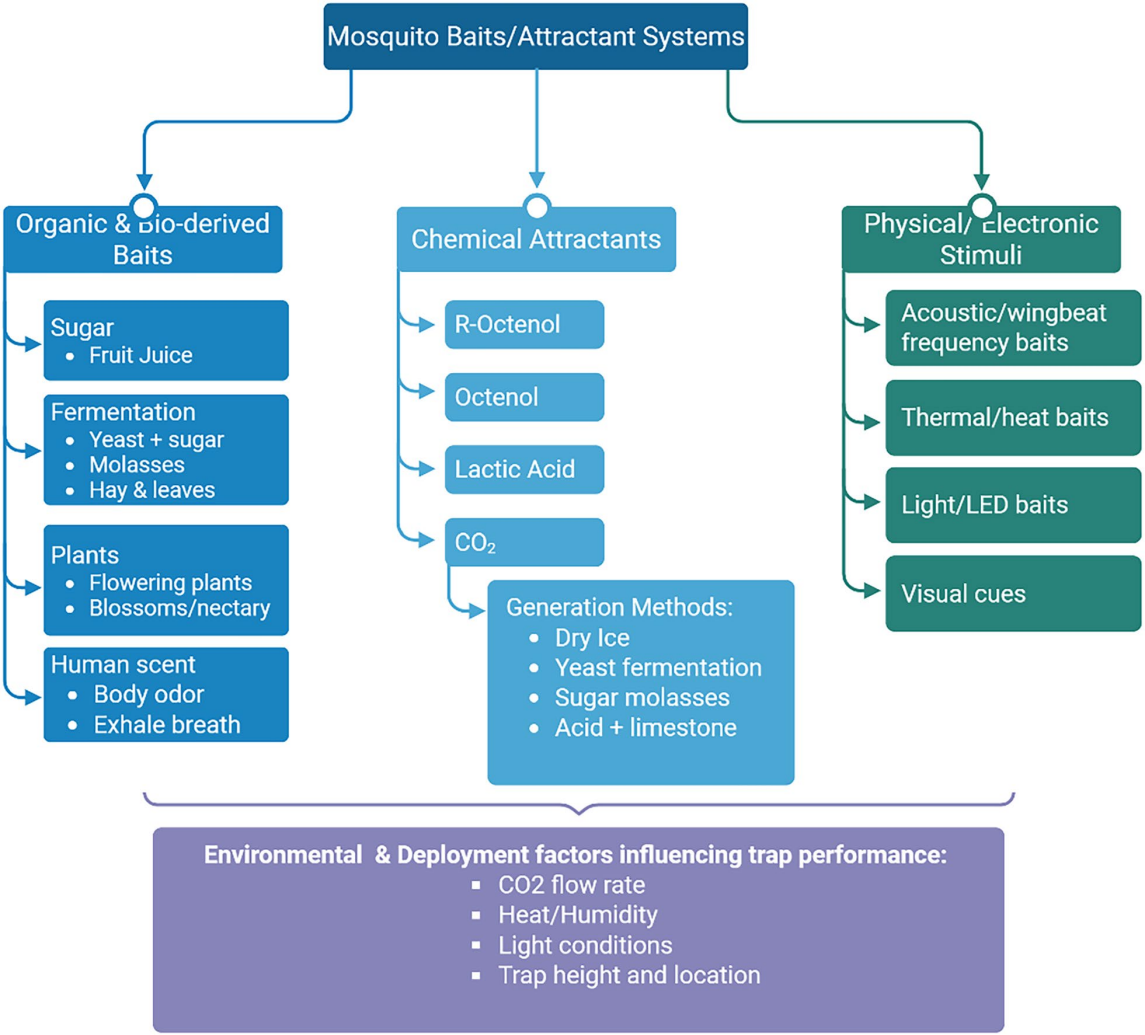
Mosquito bait taxonomy. Schematic diagram depicting the hierarchical organization of mosquito baits and attractants. At the top level, the attractants are divided into three major categories: organic and bioderived baits, chemical attractants, and physical/electronic stimuli. From each of those categories, they branch into multiple subclasses, illustrating the wide range of compounds/stimuli currently known as mosquito baits and attractants

In an effort to address what can be discussed further and how these gaps can be filled by analyzing additional research avenues, this review proposes a conceptual taxonomy of mosquito baits and attractant systems (Fig. 1) to unify the three major categories of mosquito baits. This taxonomy is constructed through a literature review of mosquito baits and the identification of major contributions in their respective fields. Organic and bioderived baits include sugar, fermentation-derived cues, and plant-derived carbohydrate sources used by mosquitoes for energy acquisition. Chemical attractants comprise volatile compounds such as octenol, R-octenol, lactic acid, and CO_2_ (generated by different methods). Finally, physical and electronic stimuli include technology-assisted baiting approaches that exploit wingbeat frequency, thermal, light, and visual signals. Moreover, several key engineering variables influencing bait performance, for example, CO_2_ concentration or flow rate, CO_2_ integration with different types of attractants, use of wavelength and irradiance, sound frequency and pressure level, heat and humidity, trap height, and so on will also be discussed.

**Fig. 2:**
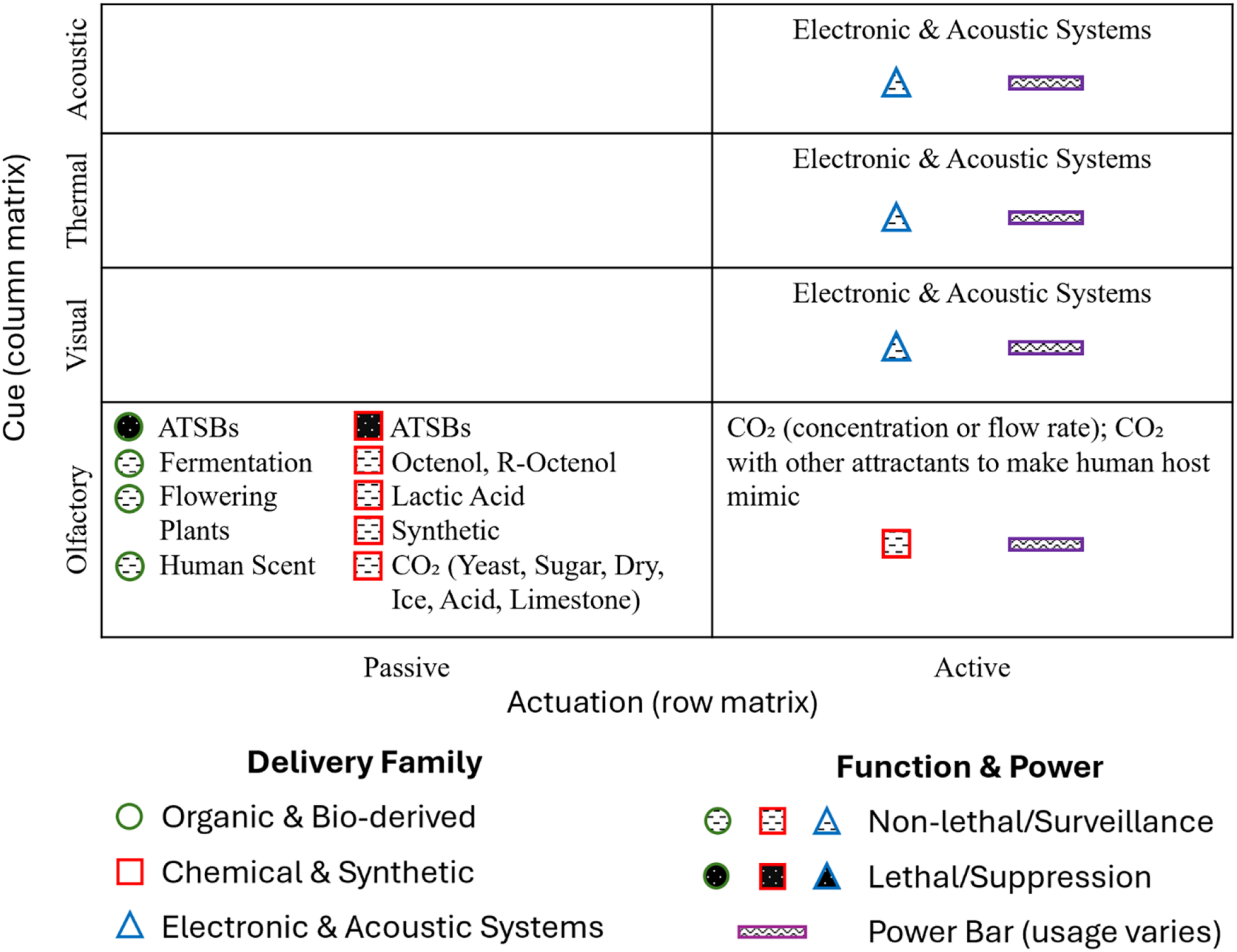
Mosquito bait fusion taxonomy matrix. Conceptual matrix organizing mosquito bait and trap strategies by actuation (passive, active, and closed-loop systems) and cues (olfactory, visual, thermal, and acoustic cues). The matrix illustrates how these combinations support different applications and highlights functional performance and power requirements. The matrix provides a unified framework for comparing technologies and possibly guides the design of the next generation of baits

To better explain the concept of this review, a fusion taxonomy matrix (Fig. 2) is proposed by organizing the baits shown in Fig. 1 into a matrix form, that illustrates the functionality, involvement of power, and nature of the cues in a cue versus actuation matrix. The cue axis (*y*-axis or column matrix) shows the nature of the baits, for example, olfactory, visual, thermal, and acoustic, and the actuation axis (*x*-axis or row matrix) shows how the baits function, for example, passively, actively, and in closed-loop. Together, they demonstrate which baits are lethal, which are nonlethal, and which are organic, chemical, or electronic. It also gives a surface-level indication of whether any power is required for any of the baits. Perspective discussions on other avenues, such as the need for closed-loop smart baits and digital twin systems, optimization challenges, different experimental approaches, ecosystem and safety, and so on, are also highlighted in this study. Collectively, these intuitive elements have the potential to establish frameworks for selective, adaptive, and energy-efficient baiting strategies.

## Mosquito baits

### What is a mosquito bait?

Mosquito baits are typically considered custom-developed tools that attract mosquitoes to a specific location, allowing them to be trapped, counted, or killed. These baits mimic the elements that naturally attract mosquitoes. These elements typically include animal scent, body heat, light of certain wavelengths, dark places, moisture, wingbeat (WB) sounds, and others. However, their efficacy remains highly dependent on mosquito species, season, and local site conditions [10–21]. Moreover, several elements are combined to make baiting or trapping more effective, and these mixtures usually depend on the mosquito species, environmental factors, location, and whether to lure or kill them. Besides, effective baiting strategies are usually crafted on the basis of an understanding of what the selected mosquito species are looking for and by avoiding the attraction of untargeted and beneficial insects such as bees.

### Traditional and novel mosquito baiting strategies and their evolution

The history of mosquito baiting dates back to the early 1900s, when live hosts and cattle were first used as baits [22]. Headlee observed that mosquitoes’ blood hunger compels them to pursue and bite humans even under challenging conditions. Roubaud further noted that mosquitoes were significantly more attracted to animals than to humans [22]. In 1910, Howlett was the first to identify temperature as a key attractant for mosquitoes [19], demonstrating that *Aedes* (*Stegomyia*) *scutellaris* were drawn to heat.

In 1922, Rudolfs first suggested that carbon dioxide could be used as an attractant [23]; Headlee (1934) was the first to discover the potential of using carbon dioxide in conjunction with a mechanical trap. He further developed carbon dioxide-baited light traps in 1941 [24].

Technology was first introduced into mosquito baiting with the early development of light traps, such as the New Jersey light trap [25]. It took advantage of the positive phototaxis of certain mosquito species by using a light source to draw them toward a collection fan. In 1966, Newhouse et al. combined carbon dioxide in the form of dry ice with the Centers for Disease Control and Prevention (CDC) trap to create a surveillance tool [24]. During the latter half of the 20th century, research in chemical ecology revealed that human skin emits key semiochemicals such as lactic acid that play a pivotal role in guiding host-seeking behavior; 1-octen-3-ol (octenol) was found to be a major component of cattle breath [26, 27]. Instead of relying solely on CO_2_, researchers created stronger, more realistic scent mixes for attracting mosquitoes by adding these compounds to baits. At the same time, hay infusions were placed in water-filled containers, where they would ferment, creating a microbial soup. These released organic compounds mimicked nutrient-rich aquatic habitats, which in turn attracted female mosquitoes [28].

During the 21st century, a “lure-and-kill” strategy was developed using attractive toxic sugar baits (ATSBs) [29]. ATSBs used a combination of attractive fruit or floral scents with a sugar solution and an oral toxicant (e.g., boric acid, spinosad) to lure both male and female mosquitoes, i.e., *An. sergentii* [30]. This enabled population control along with the surveillance of host-seeking females. At the same time, oviposition traps evolved considerably. For example, the autocidal gravid ovitrap (AGO) is a passive trap used to capture egg-laying females by utilizing water infused with decaying hay [31]. In the current era, beginning in 2010, mosquito baits have evolved toward integrating AI and machine learning with conventional baiting strategies. Modern traps utilize programmable actuators to release thermal, visual (LED), and acoustic baits, which attract mosquitoes [10, 32]. Most of these systems have different sensors and imaging platforms that enable real-time data collection across surveillance networks, thereby making the baits more efficient and precise. This evolution represents a shift from static baits to dynamic data-driven baits.

### Classification and application of mosquito baits

#### Organic and bioderived baits

Organic and environmental fermentation baits are designed to mimic natural processes and cues that mosquitoes are statistically shown to follow. We can categorize organic bait into four main types: toxic sugar baits (processed or natural), fermented baits, plant-based baits, and human-derived attractants, such as body odor and exhaled breath.

#### Sugar-based baits

Fruit juice baits are a subcategory of processed toxic sugar baits designed to attract and kill mosquitoes by mixing toxic active ingredients, such as boric acid, with fruit juices (e.g., mango, pear, apple, guava, or banana). Both males and females of *Culex*, *Anopheles*, and *Aedes* are attracted to these sugar sources, with apple juice-based baits highly attractive to *Cx. quinquefasciatus* and *An. sinensis*, and pear juice is most effective for *Ae. albopictus *[33]. Field trials in subtropical Florida showed that eugenol (a plant-derived compound, primarily from clove oil) infused ATSBs reduced *Ae. atlanticus*, *Ae. infirmatus*, and *Cx. nigripalpus* by > 70% for up to 3 weeks [34], while encapsulated garlic oil proved effective against *Ae. albopictus* in tropical regions [35]. However, nontarget effects can occur when ATSBs are applied to flowering vegetation, as pollinators and other nectar-feeding insects may ingest toxic compounds while being drawn to the floral cues [35]. However, applications on nonflowering plants show minimal impact, highlighting the need for careful deployment.

#### Fermentation-based baits

Fermentation-based baits use yeast and sugar mixtures to generate biogenic CO_2_ and volatile byproducts that mimic host cues. Carbon sources such as molasses, jaggery, or glucose are fermented by commercial yeast strains to produce continuous CO_2_ in CDC light traps or BG-Sentinel traps [36]. Yeast-based CO_2_ effectively attracts multiple species, including *Anopheles stephensi*, *Culex quinquefasciatus*, and *Aedes albopictus* [33, 36]. Yeast-sugar fermentation provides a cost-effective CO_2_ source, although its efficiency declines over time, and release is slower than that of dry ice. In contrast, alternatives such as dry ice or chemical systems (e.g., HCl dripping onto limestone) are more expensive and less field-portable, but they offer a steadier, longer-lasting output [37].

#### Plant-based baits

Plant-based baits include flowering plants, nectarines, hay, and leaf infusions. Infusions of mango, banana, cashew, or neem leaves stimulate oviposition in pregnant *Aedes*, while flowering plants such as *A. macrostachya* have been shown to surpass fruits like guava in attracting *Anopheles gambiae* [38]. These cues rely on volatile compounds and carbohydrates essential for mosquito reproduction [38].

#### Human scent baits

Human-derived attractants primarily rely on body odor or exhaled breath. Volatile compounds from skin decomposition and CO_2_ emissions serve as powerful lures for female mosquitoes, while human breath provides concentrated CO_2_ and synergistic volatiles that enhance attraction, especially for *An. arabiensis* and *An. gambiae* [39].

Collectively, organic baits are ecofriendly and relatively low-cost. ATSBs are effective at reducing populations through the “attract-and-kill” principle, but raise concerns about potential impacts on nontarget insects. Fermentation-based baits offer a feasible source of CO_2_, although their performance declines over time. Plant-based baits provide strong natural cues and can attract mosquitoes, yet their effectiveness varies by species and season. Human-mimic baits capture the most host-specific responses but are logistically complex and less practical for routine deployment. Together, these approaches hold significant promise for integrated vector management, but further optimization is necessary to balance efficacy, stability, and ecological safety. It is also crucial to consider the target species, as bait performance can differ substantially across mosquito species.

### Chemical and synthetic baits

#### CO_2_-based baits

Chemical baits are widely explored by researchers because of their potential in mosquito monitoring and control. Among them, CO_2_, used alone or in combination with other chemicals, accounts for the largest share of chemical bait studies. While CO_2_ can be produced biologically through yeast fermentation (classified as organic and bioderived baits), the CO_2_ discussed here refers to the industrial or synthetic form released from gas cylinders or dry ice, commonly used in chemical bait formulations. Field experiments in China demonstrated that Biogent (BG) traps releasing CO_2_ at 500 mL/min achieve the highest captures of *Aedes albopictus*, while both lower and higher flows reduce efficiency, demonstrating the importance of optimizing release rates [40]. Environmental variables play a key role in CO_2_ trap performance. Research from the Great Salt Lake area suggests that heat and light have a minimal influence on mosquito attraction, whereas higher humidity and temperature are associated with increased nightly captures. Trap height, in contrast, has a negligible effect [41]. Overall, CO_2_ is the primary active ingredient of attraction, with abiotic conditions contributing secondary effects.

#### Synthetic mimics

To better mimic humans, CO_2_ is often paired with odor lures. In Queensland, traps baited with CO_2_ plus either BG-Lure or natural human odor capture significantly more *Anopheles farauti* than CO_2_ alone, while the MB5 blend (a synthetic odor blend used as a lure component) is less effective [42]. These results highlight the combined effect of CO_2_ and odor, although responses vary by species. Synthetic mimics of natural plant and floral cues have also been studied as mosquito attractants. Laboratory trials using plant-based synthetic blends, such as (*E*)-linalool oxide (LO) with other chemical mixtures, demonstrate effective attraction on *Anopheles* species. Combining LO with worn socks enhanced trap captures, whereas pairing LO with a synthetic animal/human odor blend reduced trap efficiency, indicating complex odor interactions between plant- and host-derived cues [43]. Complementary experiments with flower-mimic setups of various petal colors (blue, red, yellow, pink, purple, and white) reveal that visual floral cues influence foraging and feeding behavior [44], underscoring the potential of synthetic plant and flower mimics to advance sugar-bait technologies and vector surveillance strategies.

#### ATSB-based baits and other chemical attractants

While bioderived ATSBs use natural ingredients such as plant extracts or fermentation products, chemically formulated ATSBs incorporate synthetic attractants or additives to enhance surveillance and virus detection. Beyond CO_2_, other chemical attractants have also been tested. Scented attractive toxic sugar baits (ATSBs) are designed to improve sugar-feeding surveillance. Phenyl acetaldehyde–scented sugar baits detected West Nile virus earlier than sentinel chickens in California, but effectiveness varies by site and deployment costs were higher than unscented ATSBs. Thus, unscented ATSBs are more practical for control, while scented formulations are valuable for arbovirus tracking [45]. Other chemical attractants, such as octenol, R-octenol, and lactic acid, have been evaluated but demonstrated limited effectiveness. Russell et al. reported that adding octenol to CO_2_ does not increase trap captures of *Aedes aegypti*, *Aedes polynesiensis*, or *Culex quinquefasciatus* and, in several cases, even reduces trapping efficiency [46]. Similarly, R-octenol does not improve *Ae. albopictus* catches compared with BG human-skin lures [47]. Lactic acid also fails to enhance CO_2_-based attraction, further confirming the limited contribution of these compounds to mosquito trapping performance.

Certain toxicants serve as bait enhancers. Boric acid, used in ovicidal traps, effectively reduces *Aedes* (*Ae. aegypti* and *Ae. albopictus*) egg-laying and survival, making it a low-cost complementary tool in integrated strategies [48]. Synthetic odor blends are also emerging as scalable alternatives. BG-Lure with CO_2_ performed comparably to natural human odor for attracting *Anopheles farauti* [39] . Plant-based blends such as linalool oxide and ocimene attracted *An. gambiae* and *Anopheles funestus* at rates similar to human-derived odors, with or without CO_2_, underscoring their potential in malaria surveillance [43].

In summary, CO_2_ remains the focus of chemical baits owing to its consistent attractiveness across species. Scented sugar baits and synthetic blends expand applications, but additives such as octenol and lactic acid add little value. Future research should prioritize stabilizing CO_2_ release systems and refining synthetic odorants to balance cost-effectiveness, specificity, and ecological safety.

### Advancements in electronic and acoustic baiting systems

The organic and chemical baits have been around for a long time, and scientists have been widely using them for decades to study mosquito behavior and to trap, monitor, or control mosquitoes. However, several of them have adverse effects on the ecosystem and may not be very effective owing to changes in environmental factors, such as temperature, humidity, wind, and rain. In an effort to minimize adverse effects on the ecosystem and to bypass the effects of environmental factors, scientists focused on mosquito wingbeat (WB) sound, light, and heat and decided to use their artificial electronic versions as baits. However, it is important to note that these artificial electronic versions of these baits may not be as effective as their bioderived variants. A few studies have shown that temperature variation can play a significant role in *Ae. aegypti* to wingbeat sounds [48–50]. Several controlled experiments, such as lab tests, show that the female wingbeat frequency (WBF) increases by about 8–13 Hz per degree as the temperature rises from 18 to 31 °C, and it means that their sound range falls between 380–560 Hz [51]. Field tests using the MAST confirmed that wingbeat sounds released on the basis of temperature can help attract more males, for example, playing sounds around 60 dB during the day can attract as many male *Ae. aegypti* as the other top-performing baits [52]. Several experiments showed that playing sounds around 550 Hz was more effective at attracting *Ae. aegypti* and *Ae. albopictus*, while sounds with lower tones attracted more *Culex* than with the higher tones [53]. These results highlight that playing baiting acoustics with the right WBF can help in attracting more target mosquito species and reduce bycatch. Moreover, there have not been many studies that strongly support the influence of environmental factors on the WBF as bait. Although there has not been a significant amount of research done in this area compared with other types of baits, discussing what has been done so far is worth doing.

#### Influence of acoustic/wingbeat frequency baits

Laboratories are increasingly using mosquito senses to enhance bait efficacy. A significant area of research is the auditory system of mosquitoes. Acoustic baiting is an ecofriendly control method that either lures mosquitoes into traps or into desired target areas. It emits sounds that mimic the wingbeat frequency of mosquitoes. The mosquitoes sense this through the Johnson’s organ, a sensory organ present in the second segment of the antenna, and it is known to be the most complex mechanosensitive organ found in mosquitoes and other insects [49]. Steele and McDermott describe how this sensory organ functions, emphasizing that male mosquitoes respond better to moderate sound pressure levels (SPL) owing to their greater sensitivity toward sound [50]. Their review compiles wingbeat frequency (WBF) data from different mosquito species and underscores the need for clearer engineering standards to design traps on the basis of WBFs. These standards also include the frequency and duration of the sounds played by factoring in temperature changes.

Mosquito mating happens midflight, mediated by the harmonization of the male and female flight tones or sounds generated by their wingbeats [54, 55]. Researchers are starting to take advantage of this mating technique to attract mosquitoes with multimodal attractants [56]. Studies have demonstrated the effectiveness of frequency of wingbeat sounds in the 500–650-Hz range to attract male *Ae. albopictus* mosquitoes [57, 58]. At moderate volumes of 75–79 dB, slow sweeping performs better than playing a single tone [57]. Field tests using WBF bait-driven traps such as theMale *Aedes* Sound Trap (MAST), a specialized trap that mimics the WBF of female *Aedes* mosquitoes to lure males, found that tones around 650 Hz caught more males than sounds outside this range [58]. Younger males aged 3–5 days old are most responsive, and the response fades away as they age [46] . These studies propose that with the right range and volume of sound and dark or shaded spots catch more *Ae. albopictus* males [57, 58].

#### Use of acoustics/wingbeat sounds in microcontroller-based baits

Two recent studies have shown the usability of mosquito wingbeat sound research into practical tools [59, 60]. Johnson et al. used an Arduino microcontroller to create a 484 Hz female *Ae. aegypti* mosquitoes tone inside a passive (nonpowered) gravid *Aedes* trap (GAT) to lure and capture males [59]. Field trials demonstrate higher male lures and capture than nonbaited and mains-powered ones, but at a much lower cost [59]. It should be noted that it is a simple open-loop non-AI system that works well for routine monitoring in mosquito release programs [59]. In another study, Ramkaow et al. utilized an ESP32 microcontroller with microphones to identify mosquito wingbeat sounds of 300–700 Hz, and when it hears the right sound, it turns on a high-voltage grid to activate bait materials, such as CO_2_ and an ultraviolet (UV) suction fan, and kill the oncoming mosquitoes [60]. However, this identification and baiting system only works for a short range of 15 cm and can be affected by the sound of the wind [60]. It is only a closed-loop system in the sense that it turns on power when it hears the targeted wingbeat sound [60].

#### Wingbeat sound collection strategies

Building on the concept of wingbeat frequency-driven attraction, the following sections detail the experimental methods and novel technologies employed in this research involving data collection strategies.

##### Field testing

Data collection of wingbeat frequencies of mosquitoes is the primary step in being able to take advantage of the mosquito auditory system for bait. One way researchers are gathering wingbeat frequencies is by recording wild mosquito swarms in the field with particle velocity and pressure-sensitive microphone arrays [55]. The downside to gathering data in the field is the interaction of the Doppler effect and other environmental sounds can diminish the signal-to-noise ratio of the mosquito wingbeat [56]. The benefit of field testing is that it yields the most realistic sounds that mosquitoes interact with.

##### Laboratory testing

To focus on the wingbeat frequencies isolated from the environment, other methods of recording wingbeats have been researched. Controlled recordings to isolate auditory signals from mosquitoes fall into two categories: tethered or free-flight mosquitoes [61, 62]. Tethered mosquitoes are connected to restraining structures that mitigate their movement and thus allow recording devices near the mosquitoes to gather signals with a high signal-to-noise ratio [61, 62]. Furthermore, free-flight mosquitoes monitoring in laboratories records unmitigated mosquitoes in acoustically dampened environments, allowing the collection of natural flight stimulus and data [63]. The significant findings from this research are that mosquitoes have sex-specific sensitivity to wingbeat frequencies and circadian patterns of responsiveness [10]. The benefit of these studies is that they allow for precise characterization of artificial lure design, but they may not fully replicate field conditions.

#### Acoustic/wingbeat sound database

Three complementary mosquito acoustic (wingbeat frequency or WBF) resources have been developed to address their distinct needs in mosquito identification and control [64–66].

HumBugDB is a large, compiled database containing 20 h of labeled mosquito wingbeat/flight tones from 36 species with 15 h of background noise [56]. The recordings took place in the USA, UK, Kenya, Thailand, and Tanzania using smartphones and high-quality microphones with sampling rates of 8 kHz and 44.1 kHz [64]. The repository includes tutorials and machine learning models for detecting and identifying 36 species, which makes it ideal for building IoT-based mosquito detectors [64]. HumBugDB also includes uncertainty estimates and matched negative samples to help improve accuracy and reduce false alarms [64]. MACSFeD is a temperature-aware database built from published studies with male attraction responses and wingbeat frequencies for different species and sexes [65]. It is suitable for studying different baits on the basis of mosquito response to temperature changes [57]. However, it does not include tutorials or machine learning tools [65]. The Figshare dataset offers lab-recorded mosquito wingbeat/flight tones of three species at sampling rates of 8 kHz, 44.1 kHz, and 48 kHz [66]. However, it has a limited number of species and does not include negative samples or background noises [66]. In short, if someone wants to do a comprehensive study on mosquito WBF and its effect as lures and wants to build a mosquito detector from scratch, then HumBugDB [64] is an ideal choice.

#### Integration of acoustic and visual baits

Recent research developments have deepened our understanding of the mosquito response toward the combination of acoustic and visual baits [67, 68]. For example, male *An. coluzzii* species utilize 450 Hz female wingbeat sound to help them visually track and avoid bumping into other female species in a swarm, known as acoustic gating, showing the effectiveness of the combination of acoustic and visual cue signals [67]. Building on these findings, Jakhete et al. showed that there is an increased *Ae. aegypti* male mosquito captures when a frequency-modulated chirp is combined with a high-contrast black visual cue [68]. Similarly, Balestrino et al. showed in their controlled lab experiments that when 500–650 Hz of sweeping WB sounds are paired with black visual tubes, they improve the attraction of *Ae. albopictus*; however, they suggest fine-tuning these traps before outdoor deployment [57]. Steele and McDermott also highlight the importance of visual cues in trap designs to bait mosquitoes; for example, matte-black designs and swarm-like markers are more attractive to mosquitoes [50]. These studies highlight the influence of combinations of multiple types of cue signals, especially dynamic WBF sounds combined with string visual contrasts, to build better mosquito baits.

#### Effect of thermal and light baits

Several of the recent studies have helped in identifying some of the good thermal and visual-based mosquito baits [41, 69–72]. By contrast, several of them do not; for example, it was shown in a study that cool-white light-emitting diodes (LED) used with CO_2_ baits reduced the number of *Aedes* mosquito catches [41]. Moreover, a 135 °C heat source and an incandescent bulb used with CO_2_ baits had inconsistent and little effects [41]. Therefore, the research recommendation was not to add light sources to avoid bycatch but to keep CO_2_ baiting systems simple [41]. Motivated by this, another study found that Mosclean UV-LED-based baits with a wavelength of 365 nm, without heat or CO_2_, attracted more *An. arabiensis* and *Culex* mosquitoes than the CDC light traps [69]. Another study developed and tested a solar-powered UV bait prototype with different wavelengths and optical lenses and reflectors and found out that the design performs well off-grid; however, it did not measure CO_2_ or heat and lacked proper controls [70]. However, “Silva,” a passive LED trap, captured as many *Anopheles* mosquitoes as the CDC trap over 12 nights using less power, but it was tested in one location without the support of any other baits [71]. In another study on the comparison of different colors of light, it was found that UV fluorescent light and UV-LED were more effective as cues than blue, green, and red lights [72]. However, they did not measure the intensity of light but identified peak activity mosquito times [72]. All of these findings indicate that several light baits themselves can be effective to lure mosquitoes, but they should be avoided when used with CO_2_.

#### Multimodal bait development using acoustic/wingbeat cues

A promising outcome from mosquito wingbeat collection is the development of multimodal baits that combine acoustic cues/baits with other sensory stimuli. Recent studies have begun to integrate flight tones with olfactory signals, such as CO_2_ and human odor, as well as visual stimuli, to enhance trap effectiveness [73, 74]. For example, traps that emit female WB sounds or flight tones have been shown to attract male mosquitoes, demonstrating the potential for sex-specific targeting [62]. Evidence suggests that incorporating acoustic cues can increase both the efficiency and selectivity of traps. However, challenges remain in optimizing these systems, particularly in tuning the traps to attract specific species or sexes without inadvertently affecting nontarget organisms.

### Behavioral responses of mosquitoes toward baits

Mosquito behavioral responses to bait can be grouped into four main categories: feeding, host-seeking, mating, and oviposition. Feeding behavior is elicited by organic, bioderived, and chemically derived sugar-based baits, including toxic sugar baits, scented sucrose blends, and synthetic nectar mimics. These baits trigger nectar foraging responses involving rapid orientation, hovering, and probing, enhanced by floral or fruit volatiles [35, 75, 76]. Host-seeking behavior is driven by fermentation-based baits that release CO_2_ and other volatiles, as well as chemical CO_2_ baits and human-derived attractants that emit heat, moisture, and body odors [77–79]. These cues provoke upwind flight, zigzag orientation, and repeated probing similar to natural host-tracking [80, 81]. Mating behavior is exploited by using acoustic baits that reproduce female wingbeat frequencies, prompting males to swarm, hover, and orient toward the sound, often paired with light or CO_2_ [82, 83]. Oviposition behavior is influenced by plant-based baits and visually optimized traps, especially black ovitraps, which attract gravid females [84, 85]. This leads to skimming, abdomen dipping, and egg-laying [86]. Environmental factors play a key role in mosquito behavior. For example, mosquitoes live longer and reproduce more in sugar-rich areas, and female mosquitoes tend to bite more often in areas with less sugar; that is why nectar-based lures are considered suitable in mosquito control strategies [87].

Altogether, organic, chemical, and electronic baits strategically manipulate these innate behaviors to improve mosquito attraction, monitoring, and population control. However, owing to these distinctive behaviors, different traps may introduce sampling biases into surveillance collections, including age structure, physiological status, and sex. These biases have important implications for the interpretation of surveillance data and estimates of mosquito population dynamics. With that in mind, trap selection should take surveillance objectives into account while acknowledging inherent biases to ensure accurate and meaningful data interpretation. Practical considerations related to specimen collection and preservation should also be taken into account, as different traps may capture mosquitoes dead or alive, intact or damaged, which can influence downstream analyses (e.g., morphological identification, pathogen detection, and molecular studies). Therefore, trap choice should also reflect the subsequent analyses planned for the collected specimens.

### Fundamental dynamics of human-mosquito interactions involving different attractants

Mosquitoes usually follow a step-by-step process to locate and feed on human blood; they do not randomly smell people and bite them. When humans exhale, even with a brief puff, they release CO_2,_ which triggers the mosquitoes, i.e., *Ae. aegypti,* to become more responsive to movement and contrast by switching into a visual search mode [88]. Color plays an important role for mosquitoes in locating hosts, i.e., humans, after their smelling sensor is activated with the exhaled CO_2_, and with that activation, they become more visually alert [84]. But what is interesting is that while their visual sensitivity can be boosted by smell, their sense of smell does not get stronger after seeing the same thing. Once activated, they track humans using microbiome and skin-derived cues such as ammonia and lactic acid [89]. As the mosquitoes get closer, they rely on a few additional factors to decide where to land and bite, such as humidity, human skin contrast against the background, heat differences, and how the skin feels [89, 90]. They are usually drawn to darker colors and high-contrast objects [84]. This complex behavior is influenced by multiple sensory systems, such as temperature receptors, proteins such as opsins, a CO_2_-sensitive neuron called cpA, and TRP channels that detect heat and light [89, 90]. Disrupting just one of the sensory systems, for example, the ability to detect CO_2_ or colors with long wavelengths, may reduce their tracking ability to locate humans, but it does not stop them completely [84, 89, 90].

Demographic factors determine the attractiveness of humans to mosquitoes through physiological changes in the body. For example, pregnancy attracts more mosquitoes owing to increased exhalation of CO_2_, raised skin temperature, and changes in released body odor [89]. Humans with faster metabolisms and larger bodies tend to attract more mosquitoes [89]. Age, gender, or ethnicity do not have steady effects on attractiveness; however, the unique blend of the chemistry of human sweat and bacteria on a person’s skin plays a better role in attractiveness [87–91].

Odor cues also influence their color preferences; for example, floral scents increase their attraction toward green color, and the smell of human feet makes them attracted toward a wide range of colors [91]. The surroundings have an impact too; shaded areas with little wind and dark objects against a bright sky make it easier for them to locate their targets, i.e., humans [84]. Different species have a fondness toward different colors; for example, *Aedes* mosquitoes are attracted toward warm colors such as red and orange, *Culex* mosquitoes tend to go for blue and red, and *Anopheles* mosquitoes are drawn to black or red [84, 87]. Therefore, the mosquito’s attraction to humans also varies depending on the colors they wear or the colors around them.

For real-world applications that lure or trap mosquitoes, devices should incorporate a dark or high-contrast target, localized heat, short puffs of CO_2_, or different human mimics and moderate humidity. To keep mosquitoes from getting adapted to these signals, duty cycles and device placement are recommended to prevent sensory adaptation and maintain efficacy [87–91].

### Experimental techniques and instrumentation in mosquito bait research

Experimental studies of mosquitoes rely on a multiscale approach, progressing from controlled laboratory assays to semifield and field deployments. This methodological pipeline allows researchers to isolate behavioral responses to specific stimuli before evaluating their effectiveness under more realistic ecological conditions. This section outlines the key techniques and instrumentation commonly used in mosquito bait research.

### Controlled laboratory assays

Laboratory-based assays provide data for bait development by assessing mosquito behavioral responses in tightly controlled environments. Here are a few techniques used in a lab environment.

#### Olfactometers

Olfactometers provide controlled environments for quantifying mosquito responses to chemical stimuli. In multi-arm olfactometer setups such as the four-arm device used by Xie et al. to study *Aedes albopictus*, mosquitoes are released in a central chamber and presented with multiple airflow channels, each carrying a distinct odorant or blend [92]. Their distribution among the arms provides a quantitative measure of preference, enabling researchers to simultaneously compare several candidate compounds against controls. The controlled airflow ensures reproducible odor delivery, isolates olfactory stimuli from other cues, and facilitates robust statistical analysis of behavioral responses. By contrast, the uniport olfactometer focuses on a single odor stimulus, presented at one end of a release chamber, with mosquitoes at the opposite end [93]. The design ensures that only olfactory cues drive attraction, allowing researchers to precisely measure the proportion of mosquitoes responding to a specific compound or blend. This high degree of reproducibility makes the uniport olfactometer a valuable tool for standardizing behavioral assays and isolating the effects of individual odorants. While the classic Y-tube olfactometer remains a standard for assessing spatial repellency or attractant preference in a binary choice paradigm, modern systems have evolved into multi-chamber apparatuses. These systems could be designed for precise environmental regulation (e.g., temperature ± 0.15 °C, humidity ± 2%), and the ability to incorporate automated tracking and counting to eliminate observer bias [94–96].

#### Wind tunnels

To study more complex host-seeking and other flight behaviors, researchers utilize climate-controlled wind tunnels. These systems can be designed to simulate specific atmospheric conditions, with adjustable CO₂ concentrations (from ambient up to 4,800 ppm), temperature (e.g., 27 °C ± 0.1 °C), and humidity (e.g., 70% ± 5%). They can also be equipped with synchronized high-speed cameras (90 fps or higher), enabling flight path analysis and providing insights into how mosquitoes navigate odor plumes from stimuli such as human odor or synthetic chemical blends [97, 98].

#### Tunnel and cage assays

To assess the terminal efficacy of a bait or treated surface (e.g., blood-feeding inhibition and mortality), standardized tunnel tests are used. These assays, which often involve 60-cm glass tunnels with treated netting barriers, offer a safe and epidemiologically relevant alternative to human landing catches. Mosquitoes are granted controlled access to an animal bait over a 12–15-h period, allowing for the quantification of key performance outcomes under standardized conditions (27 °C ± 2 °C and 75% ± 10% RH) [99].

### Semifield and field-simulated systems

To validate laboratory findings, research moves to semifield systems that introduce environmental realism while maintaining experimental control.

Greenhouse-enclosed semifield environments, such as the MalariaSphere, are specifically designed to facilitate realistic and contained research on mosquito vectors and vector control tools [100]. This greenhouse structure incorporates features of the *An. gambiae*’s natural ecosystem, containing local vegetation, breeding and resting sites, and a traditional village house, all under greenhouse cover. Climate conditions within the facility closely mimic those found in the surrounding region, creating microclimatic conditions, such as humidity, light cycles, and temperature, that parallel those of the external environment while dampening extreme fluctuations. This careful design allows researchers to conduct studies on mosquito behavior and life-cycle processes, including host-seeking, mating, oviposition, and resting under conditions that resemble the wild while providing the safety and rigor of containment.

A key advantage of such a greenhouse-enclosed system is the ability to precisely manipulate environmental variables and bait types. For example, within the MalariaSphere, bait design and deployment can be evaluated in behavioral assays that use physical traps, dry ice and protected human volunteers as standardized attractants. Notably, this setup enables direct assessment of behavioral endpoints central to bait evaluation, such as trap entry rates, attraction profiles, and avoidance behaviors, offering valuable insights before deployment in uncontrolled field settings.

### Advanced field instrumentation and data analytics

The effectiveness of baits in the field is increasingly being measured using a suite of electronic sensors and data transmission technologies that enable automated, large-scale monitoring.

#### Smart traps and automated surveillance

Modern traps are evolving into “smart” devices. Species identification is being automated using acoustic sensors that detect species-specific wingbeat frequencies (200–1,000 Hz) or, more robustly, optical infrared sensors that are immune to background noise [101]. Onboard computer vision systems, using convolutional neural networks (CNNs), can now achieve high accuracy (e.g., 92%) in distinguishing key vector species [32]. Integrated with telemetry (via cellular or other networks) and GPS, these smart traps create a network for transmitting real-time, geolocated population data without manual intervention [102].

#### Environmental contextualization

Trap capture data is critically dependent on local environmental conditions. To account for this, researchers employ sensors that simultaneously monitor CO₂, temperature, and humidity and log the data. With long battery life and wireless connectivity, these systems could provide the essential environmental context needed to interpret a bait or trap’s performance on any given night.

#### Performance metrics

The maturation of the field is reflected in the development of novel metrics such as “catch-per-joule” and the use of statistical calibration tools that compare trap performance against human landing catch baselines, acknowledging that correlation strengths can vary widely (e.g., *R*^2^ from 2.5% to 53.4%) depending on the species and conditions [103, 104].

### Optimization challenges

Optimizing a mosquito bait system poses complex engineering challenges that require balancing multiple competing objectives: maximizing catch efficiency while minimizing resource consumption, adapting to environmental variability, and achieving species-selective targeting. The catch-per-dollar metric provides a standardized approach for comparing different trap configurations and optimization strategies. Research indicates that optimized systems can achieve 2.5–4.5 times higher efficiency compared with nonoptimized deployments [105].

#### Optimized trap hardware design

Recent developments in three-dimensional (3D)-printed trap designs have demonstrated equivalent performance to commercial systems while enabling customization for specific deployment scenarios. The Salt Lake City (SLC) trap showed statistically equivalent species diversity and catch efficiency compared with commercial alternatives, while offering significant cost reductions and supply chain independence [106].

#### Power optimization

Energy consumption is a primary limiting factor for deploying remote, autonomous mosquito monitoring and baiting systems. Traditional designs often prioritize catch rates over power efficiency, leading to systems that require frequent charging cycles or high operational costs. The current consensus is that CO_2_ is a more effective attractant, with traps using CO_2_ alone showing significantly higher detection probabilities for *Ae. aegypti* and Cu. *quinquefasciatus* compared with commercial lures. However, CO_2_ generation and storage systems could consume substantial resources [37, 107]. Chemical CO_2_ generation systems can produce approximately 55 mL of CO_2_ per minute using limestone powder and hydrochloric acid, providing an energy-efficient alternative to dry ice systems. These systems achieved performance equivalent to traditional dry ice traps while eliminating the need for frequent dry ice replacement and reducing logistical challenges in remote deployment scenarios [37].

Power-aware metrics are being increasingly adopted, particularly “catch per joule” calculations that evaluate trap efficiency relative to energy consumption. Research demonstrates that PWM-driven LED systems operating at 64-Hz frequency achieve optimal energy efficiency while maintaining superior attraction capabilities [108, 109]. Power consumption analysis reveals that UV LED systems that consume only 15% of the traditional fluorescent lamp power can achieve 146% higher mosquito capture rates [41]. LED lighting systems provide substantial power savings compared with incandescent bulbs, though research indicates that LED light traps may depress the collection of key species compared with incandescent or heat-film alternatives [41]. This highlights the complexity of trade-offs in optimization, where energy-efficiency improvements may compromise capture effectiveness.

#### Adaptive scheduling and trap placement

Bayesian experimental design (BED) could improve mosquito surveillance planning by optimizing spatiotemporal sampling schedules. These approaches frame survey planning as optimization problems aimed at maximizing expected information for specific research goals, whether to understand environmental associations or to minimize uncertainty in high-risk areas. BED applications demonstrate that optimized surveys comprising 20 visits are more effective when compared with repeating schedules of 111 visits used in traditional approaches [110]. This represents significant resource savings for vector control agencies while improving data quality and spatial coverage. Bayesian optimization approaches have shown promise in agricultural pest monitoring, where trap networks targeting *Helicoverpa zea* achieved improved efficiency through learning optimal spatial distribution patterns [111]. Although the methods have shown improvement in other entomological/agricultural studies, implementation and follow-up studies have yet to be conducted for mosquito bait applications.

Multi-armed bandit (MAB) algorithms provide an ideal framework for mosquito trap optimization, as they balance exploration and exploitation under uncertainty. Contextual multi-armed bandits (CMAB) incorporate environmental variables such as temperature, humidity, wind speed, and time of day to optimize trap settings. In the context of mosquito control, CMAB systems operate through a continuous decision-making loop, where each “arm” represents a specific trap configuration or placement strategy. Unlike traditional static placement strategies, CMAB systems can continuously adapt to changing environmental conditions, species behavior patterns, and seasonal variations to maximize trap efficiency while minimizing resource consumption [112]. Research demonstrates that environmental factors significantly influence trap performance, with humidity and temperature affecting both attractant dispersion and mosquito flight behavior [98].

### Field and road map

#### Current trends in mosquito bait in research

Current research in mosquito baiting is focused on integrating artificial intelligence (AI) and data science with entomology. The aim is to create more targeted and efficient systems. AI models are being developed to identify mosquito species with the help of wingbeat frequency and visual data captured by low-cost sensors [10, 64]. Several smart traps use computer vision technology and deep learning networks such as YOLO and DNN to identify live mosquitoes in real time [10]. This allows us to replace laborious manual traps with accurate baits controlled by AI/machine learning (ML) algorithms.

Another important change is the development of smart, adjustable systems that use much less energy and materials. Instead of releasing lures all the time, researchers are now using real-world data to carefully control when to release the baits and how strong the signals, such as CO_2_, heat, and light, should be [10]. By timing these signals to match when mosquitoes are most active and the environment is just right, the system can catch more insects while using fewer resources. Auto-dispensing systems (such as IAMR) use IoT sensors to control when and how much repellent is released [10]. Several systems even transmit data to different cloud performs for remote monitoring of mosquito populations across different locations [10]. Researchers also use solar-powered traps and edge devices to attract mosquitoes, thereby saving significant energy and resources.

These trends are moving toward a closed-loop, adaptive system [10, 64]. The integration of mobile devices, E-nose systems, and AI classifiers into the baits enables real-time monitoring using environmental data [10]. The main goal is to build smart traps that use live sensor data to identify a specific mosquito species and activate the best possible mix of lures. After catching mosquitoes, the trap analyzes the results and updates its strategy using machine learning algorithms, which is either built into the edge device or connected to the cloud. The biggest challenge is ensuring these systems work reliably and perform well across different real-world conditions.

### Ecosystem and safety

Mosquito baits play a strong role in surveillance and vector control. However, their impact on ecosystems needs careful observation. A key concern is nontarget attraction, especially to pollinators and other beneficial insects that support the natural ecosystem. Studies show that environmental factors such as temperature and humidity affect bait efficiency [41], which means they may also affect nontarget attraction. Researchers and commercial products need to consider these factors in design, using measures such as selective light spectra, trap placement away from flowering zones, actuator behavior based on adaptive learning, or mesh barriers to reduce unintended captures. Another major concern is resistance. Toxicants such as boric acid in ovicidal traps can reduce *Aedes* survival [48], but repeated use of the same active ingredients or chemicals may push mosquitoes to change their behavior. Rotating ingredients, changing doses, or mixing odorants, as tested with synthetic blends that mimic human or plant cues, can help reduce this risk [43]. The effects of active ingredients on edible crops also require further study to avoid unintended consequences.

Monitoring is critical for safe deployment. Lightweight dashboards that track bait activity and catch data in real time can indicate whether traps are operating within limits, while passive fail-safe modes keep systems safe if sensors or links fail. Altogether, these measures highlight the importance of designing mosquito baits that are both effective for vector control and environmentally responsible.

### Future directions and opportunities

#### Engineering insights

The future of mosquito bait research lies in integrating diverse experimental and instrumentation technologies into predictive, intelligent systems. Machine learning models are already being used to guide precision applications [32]. The continued convergence of IoT sensor networks, real-time species identification, and machine learning could shift mosquito control from a reactive to a predictive paradigm. Commercial availability of these smart bait networks could enable comprehensive, automated surveillance that informs adaptive, species-specific baiting strategies with minimal human intervention.

As a result, smart mosquito baits are anticipated to evolve into an adaptive, closed-loop cyber–physical system (CPS) with the ability to sense, decide, and act through on-device artificial intelligence (AI). One of the most important upcoming steps should be to incorporate a high-contrast target, localized heat, and short puffs of CO_2_ with wingbeat acoustics to create a multimodal bait that can lure and identify target mosquitoes and tune hyperparameters if the identification is unsuccessful. However, before that, a simple identification method is expected to be implemented using the aforementioned lures. The next step could be to create a digital twin of the smart bait that mimics its functionality in the real world. That way, testing will speed up, and the risk of failures in the lab or field will be lower. The success of digital twins or machine learning surrogates should enable the prediction of bycatch ratios (nontarget over target catch), relative errors across temperature, and if the system is not confident, then it should be able to adjust its behavior accordingly. Regular updates based on real-world data, training with a variety of conditions (domain randomization), and frequent calibration checks are needed to make the simulations reliable.

Making the smart baits work in the real world is just as important as building them. It is crucial to deploy a fleet of these services safely and reliably, and that can be started with small test releases (canary releases) to different environments using secure software updates. There should be safety protocols to control the CO_2_ or chemical releases, the loudness of WBF acoustics, energy usage, and so on, without relying on cloud commands, but if something goes wrong, the system should automatically roll back to safe mode. At the same time, the deployed baits should have a shared way to collect and compare data, including CO_2_ flow, presence of CO_2_ in the surroundings, diel activity (diurnal or nocturnal), light brightness, power usage, weather conditions, bycatch ratios, and so on. These baits are expected to run on solar panels or long-term LiPo batteries. Moreover, it should be able to responsibly optimize the allocation of the limited energy across cues such as CO_2_ pulses, acoustic sweeps, heat or light, diel activity patterns, and so on. Digital twins may be able to forecast the weather conditions and optimize energy consumption. These baits can be designed to run independently by adjusting their behavior based on which mosquito species are nearby or approaching. The baits can choose the right cues to attract the mosquitoes, for example, using WBF acoustics and dark visuals for *Aedes* males, using CO_2_ with heat and humidity for *Anopheles*, and so on [84, 87]. Moreover, the baits should avoid using white light nearby to reduce bycatch [84].

In short, the next step is to develop a complete, closed-loop system for smart mosquito baits that work responsibly from design to deployment. Putting all the aforementioned pieces together will make the baits smarter, more targeted, and more reliable. Moreover, it will make results consistent and scalable so that results from different regions can be combined and compared effectively.

#### Molecular and genetic engineering insights

**Fig. 3:**
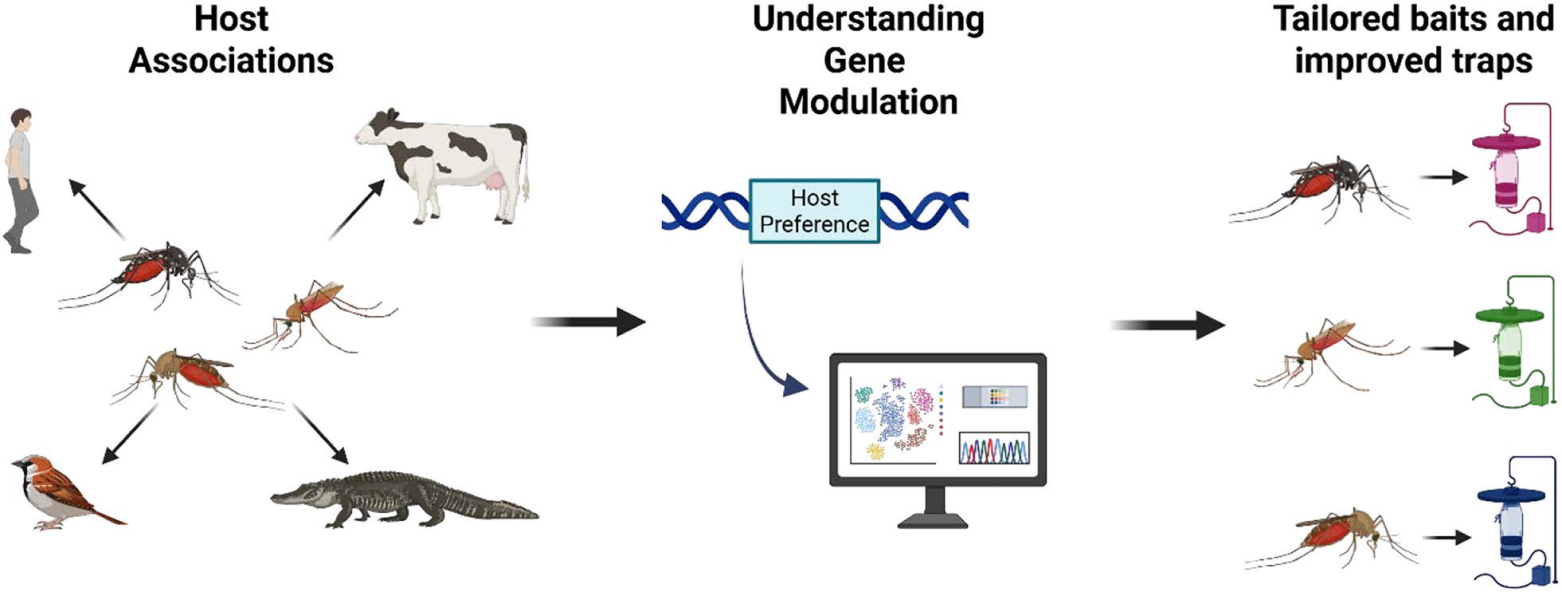
Genetic modulation of mosquito host preference and implications for development of tailored baits. Schematic representation of mosquito host associations across different species, illustrating attraction to different hosts (e.g., humans, birds, livestock, and reptiles). Host seeking and host preference are modulated by species-specific genetic factors. The identification and deep understanding of genes underlying mosquito–host interactions can be leveraged to develop tailored baits and enhance trap capabilities for specific mosquito species, ultimately improving precision, optimizing resource use, minimizing impacts on nontarget species, and increasing the efficacy of vector surveillance and control

Molecular and genomic data offer valuable insights into mosquito sensory biology and behavior, advancing the development of effective baits. The molecular investigation of sensory receptors in mosquitoes is an expanding field of research, providing insights into how mosquitoes detect and respond to diverse environmental cues that shape their host preferences. These preferences are not fixed but highly plastic, varying with host availability [113, 114]. For example, *Anopheles* females from several populations have been shown to differ significantly in host preference along a gradient from domestic (village) to sylvatic environments [113]. In mosquitoes, host preference is a trait modulated by specific genes [115–117], and it can be rapidly selected over a few generations [118]. Understanding the genetic basis of host preference, including the genes involved, the specific host cues to which they respond, and the molecular mechanisms underlying these interactions, can greatly enhance the rational design of baits. However, current baits and associated traps do not account for this variability and are often deployed uniformly across diverse ecological settings, despite differences in local mosquito host preferences, highlighting the need to reconsider the “one size fits all” approach currently used in mosquito surveillance and control programs (Fig. 3). Developing tailored baits that reflect local sensory and behavioral profiles could substantially enhance capture efficiency and improve the effectiveness of monitoring and control efforts.

Another possibility to improve bait and trapping efficiency, yet still largely unexplored, is the application of genetic engineering to mosquitoes. There are three main classes of olfactory receptors: gustatory receptors (GRs), ionotropic receptors (IRs), and odorant receptors (ORs), each with a distinct structure and role in the olfactory system [119]. Several receptors have been identified, including 131 in *Ae. aegypti* [120], 180 in *Culex quinquefasciatus* [121], and 79 in *An. gambiae* [122], but only a small fraction have been functionally characterized. With a more comprehensive understanding of the genetic and molecular mechanisms underlying mosquito olfaction, it may become feasible to manipulate genes associated with odor detection and host-seeking behavior to enhance attraction to specific baits. Such targeted genetic modifications could increase trap specificity and efficiency while reducing the unintended capture of nontarget species.

Ultimately, integrating cutting-edge engineering technologies into bait development with the latest advances in mosquito genetic engineering offers a unique opportunity to enhance mosquito surveillance and control in a more targeted and species-specific manner, with the overall goal of reducing disease transmission while minimizing environmental impact.

## Conclusions

We have developed a comprehensive review of different types of mosquito baits, organizing and structuring the existing research into different categories, including bioderived baits, chemical and synthetic baits, and electronic and acoustic baiting systems. Our focus is to make explicit the scientific insights into mosquito behavior that underpin the various baiting approaches. We have outlined the transition from lab and semifield experiments to real-world field trials and highlighted directions for more efficient and environmentally friendly baits that optimize energy use, account for species-specific responses, and minimize harm to nontarget insects. We hope the systematic organization and structure introduced in the review will help new researchers comprehend this societally critical and complex topic and develop a path toward an adaptable, standardized approach to mosquito control.

## Data Availability

Data supporting the main conclusions of this study are included in the manuscript.
